# Is the process of stabilization of carotid plaque more dynamic than expected? a high-resolution 3D-CMR statin-naive human study

**DOI:** 10.1186/1532-429X-13-S1-O96

**Published:** 2011-02-02

**Authors:** Robert WW Biederman, David R Neff, Saundra B Grant, Ronald B Williams, Geetha Rayarao, June A Yamrozik, George Angheloiu, Sobhan Kodali, Vikas K Rathi, Mark Doyle

**Affiliations:** 1 Allegheny General Hospital, Pittsburgh, PA, USA; 2 Bon Secours Heart and Vascular Institute, Richmond, VA, USA

## Introduction

Atherosclerosis is a dynamic process thought to stabilize with statin therapy. However, the uniformity of plaque stabilization and subsequent regression when examined under high-resolution 3D CMR is unknown. We hypothesize that plaque characteristics illustrate marked heterogeneity with both plaque regression *and* progression as lipid lowering therapy is instituted.

## Purpose

### Methods

Via CMR (1.5T GE), 707-two mm contiguous *in vivo* slices of advanced carotid disease (>50%; mean 64±21 ) representing 42 complete bilateral human plaques (age 68±15yrs) were analyzed for 2D/3D extent of vascular wall: lipid pool, fibrous cap, outer wall area (OWA), vessel wall area (VWA), lumen area (LA) and lipid pool (LP). All were related to fasting lipids relative to %stenosis via QPlaque (Medis). Plaque morphology was determined by CMR (T1/T2/PD) at baseline and one year following lipid lowering agent (simvastatin or simva/ezetimibe). Plaque progression was defined as LP pre/LP post < 1 while plaque regression was defined as LP pre/LP post >1.

## Results

39/42 *in vivo* plaques in statin naïve pts were successfully imaged. Resolution: 1x1x2mm. Pre therapy, mg/dL range of LDLC was 60-189 (mean 142), HDLC: 23-71 and TG: 80-214. LP represented 30±4% and fibrous laque 9±22% of total vessel wall. Post therapy, LDL was 66±31mg/dL. In 707 slices, 378 (53.5%) demonstrated progression while 329 (46.5%) showed regression. In those plaques that regressed there was significant decrease in both OWA, VWA and fibrous plaque as well as a paradoxical decrease in LA (p<0.0001 for all) while he converse was true for progression (p <0.0001 for all but LA). Specifically, LA decreased from 27.0 to 21.8mm^2^ to (19%; p<0.0001) while LA increased from 24.4 to 26.0mm^2^ (9%; p=NS). Segmenting for quartiles of LDL favorably trended with ΔLA; r=0.35, p=0.08 while lumen size was related to ΔLDL, p<0.02).

## Conclusions

In statin naïve pts, administration of lipid modulating agents appear to have initial paradoxical effects on lumen size as assessed by high-resolution 3D CMR: as VWA and LP *decreases* so does the LA. Similarly, as VWA and LP *increases* so does LA. All appears driven by the effectiveness of the ΔLDL achieved. Thus, marked heterogeneity in plaque compositional changes exist that are only resolved once ΔLDL is known.

